# NCTD promotes Birinapant-mediated anticancer activity in breast cancer cells by downregulation of c-FLIP

**DOI:** 10.18632/oncotarget.15848

**Published:** 2017-03-02

**Authors:** Li Zhao, Guoshan Yang, Hao Bai, Minghui Zhang, Dongcheng Mou

**Affiliations:** ^1^ Department of General Surgery, The First Hospital of Tsinghua University, Beijing, China; ^2^ Division of Hematology, Johns Hopkins University School of Medicine, Baltimore, MD, USA; ^3^ School of Medicine Tsinghua University, Beijing, China

**Keywords:** SMAC mimetic, norcantharidin, breast cancer, c-FLIP

## Abstract

Second mitochondria-derived activator of caspases (SMAC) mimetics is a class of new anticancer agents. However, most cancers exhibit *de novo* or acquired resistance to SMAC mimetics, posting a problem for broad applications in clinic, and highlighting the necessity of exploring combinational strategies to circumvent SMAC mimetic-resistance. We here showed that Norcantharidin, a drug that is currently being used in cancer treatment, significantly enhanced SMAC mimetic Birinapant-mediated cell viability inhibition and robustly promoted apoptosis in established breast carcinoma cell lines, as well as in primary breast carcinoma cells. Mechanistically, we revealed that Norcantharidin effectively reduced the levels of two major protein isoforms of cellular FLICE-like inhibitor protein(c-FLIP), namely c-FLIP long (c-FLIPL) and c-FLIP short (c-FLIPS). Moreover, Norcantharidin markedly enhanced Birinapant-triggered caspase-8/caspase-3 cascade. Inhibition of caspase-8 activity by a synthetic peptide Z-IETD-FMK significantly attenuated cell death induction by the combination, suggesting that caspase-8 plays a critical role in the anticancer action. In conclusion, our study suggests that the combination of SMAC mimetics with Norcantharidin represents a novel strategy in breast cancer therapy and warrants further studies.

## INTRODUCTION

Breast cancer is the most frequent type of cancer in women, and represents a major public health problem worldwide. In last few decades, several advances, such as early diagnosis and hormonal therapy, have considerably improved the general survival for patients with breast cancer [1]. Nevertheless, the prognosis for patients with aggressive or metastatic breast cancers remains poor. Hence, it is imperative to explore new therapies for those breast cancer patients [2].

Inhibitor of apoptosis (IAP) family proteins are key negative regulators of apoptosis. X-linked IAP (XIAP), cellular IAP-1 (cIAP-1) and cellular IAP-2 (cIAP-2) are among the most intensively studied family IAP members. All these three IAPs have been documented as potent endogenous inhibitors against apoptosis signaling transduction. XIAP blocks apoptosis signaling by directly inhibiting the activation of initiator caspase-9, and effector caspases-3/7, while cIAP-1/2 are involved in negatively regulating death-receptor-mediated caspase-8 activation through interaction with Receptor-Interacting Protein (RIP1). CIAP-1/2 also play important roles in regulating tumor cell survival pathways such as Nuclear Factor kappaB (NF-κB) signaling [3, 4]. These IAP family members are significantly up-regulated in breast cancer tissues and cancer cells, and are negatively correlated with patient survival. As such, targeting IAPs is a promising therapeutic strategy for breast cancer [3].

The antiapoptotic function of IAPs could be neutralized by Second mitochondria-derived activator of caspases (SMAC) protein [5]. Small molecules mimicking the proapoptotic function of cellular SMAC protein are a kind of novel anticancer agents. Like SMAC protein, these small molecules are able effectively to target cellular IAP-1/-2 (cIAP-1/-2) for degradation and also inhibit the antiapoptotic function of X-linked IAP (XIAP). These inhibitory effects on IAP proteins by SMAC mimetics leads to TNFα-triggered activation of caspase-8 and caspas-3 cascade and thus inducing apoptosis in cancer cells which secrete TNFα in an autocrine fashion [6, 7]. Since the advent in 2004, numerous SMAC mimetics have been synthesized for anticancer drug development. Birinapant (TL32711) is a dimeric SMAC mimetic. Preclinical studies revealed that Birinapant has potent antitumour activity in a range of cancers, and also has favorable pharmacokinetic/pharmacodynamic (PK/PD) properties. Therefore, Birinapant is being tested in clinical trials for the treatment of cancer patients. Nevertheless, like several other SMAC mimetics drug candidates, Birinapant had weak or modest single-agent antitumor activity in clinic although it effectively suppressed the expression of IAP proteins in tumor tissues [8–10]. As a result, considerable research emphasis is currently being placed on identifying optimal combinations to improve SMAC mimetic-based cancer therapy [8–10].

Norcantharidin (NCTD) is a naturally occurring plant derived compounds that is routinely used to treat patients with cancer in China [13–15]. Previous studies have revealed that NCTD potently inhibited the expression of several oncogenic proteins [11–13]. Notably, a recent study reported that NCTD suppressed the expression of cellular FLICE-like inhibitor protein (c-FLIP) in esophageal cancer cells [14]. As endogenous inhibitors of caspase-8 activation, two major protein isoforms of c-FLIP, namely c-FLIP long (c-FLIPL) and c-FLIP short (c-FLIPS) have been recognized as critical negative regulators for SMAC mimetics-triggered extrinsic apoptosis signaling [15, 16]. We therefore investigated whether NCTD could promote Birinapant-mediated anticancer activity through inhibition of c-FLIP in breast cancer cells.

## RESULTS

### Birinapant inhibits the expression of IAPs in breast cancer cells

**Figure 1:**
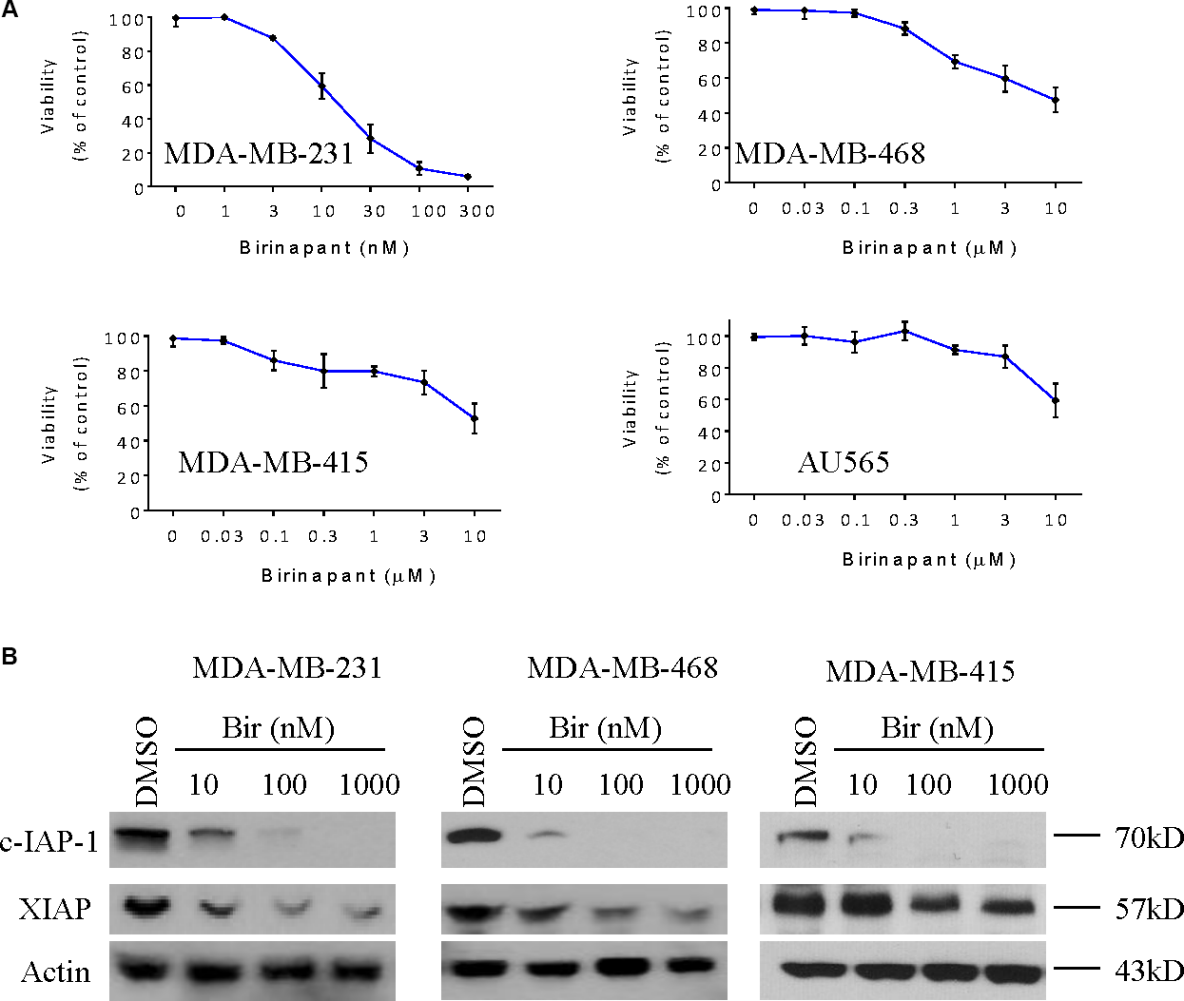
Birinapant inhibits the expression of IAPs in breast cancer cells (**A**) Human breast cancer MDA-MB-231, MDA-MB-468, MDA-MB-415 and AU565 cell lines were treated by SMAC mimetic Birinapant for 48 h, cell viabilities were determined by CCK-8 assay. (**B**) Breast cancer MDA-MB-231, MDA-MB-468, MDA-MB-415 cell lines were treated by SMAC mimetic Birinapant for 48 h. Cells were harvested and cell lysates were examined for the expression of c-IAP-1 and XIAP by western blotting analysis. Actin was used as a loading control. The data are representative results of three independent experiments.

We first tested the activity of Birinapant in a panel of 4 human breast cancer cell lines, including MDA-MB-231, MDA-MB-468, MDA-MB-415 and AU565 cell lines using CCK-8 cell viability assay. Consistent with numerous previous reports showing that MDA-MB-231 cell line belonged to the most SMAC mimetic-sensitive cell lines [7–9, 16–19], we found that Birinapant potently inhibited the viability of MDA-MB-231 cell line, with an IC50 value of 15 nM after treatment for 48 h. In MDA-MB-468 cell line, treatment with Birinapant at 10,000 nM for 48 h only inhibited cell viability by 40%, and treatment for longer time did not increase the activity of Birinapant (Figure 1A and Supplementary Figure 1A). Moreover, Birinapant also only slightly inhibited cell viability in other two cell lines. These results suggest that MDA-MB-468 and these two breast cancer cell lines are resistant to Birinapant. We next examined the effect of this SMAC mimetic on the expression of IAPs in MDA-MB-231, MDA-MB-468 and MDA-MB-415 cell lines. Western blotting analysis showed that Birinapant at 10 nM was able to reduce the level of c-IAP-1 in MDA-MB-231 cell line, and at 10 nM reduce the c-IAP-1 to minimal level in MDA-MB-468 and MDA-MB-415 cell lines (Figure 1B). Birinapant also inhibited the expression of XIAP, albeit to less extent than the inhibition of c-IAP-1.

### NCTD represses the expression of c-FLIP in breast cancer cells

**Figure 2:**
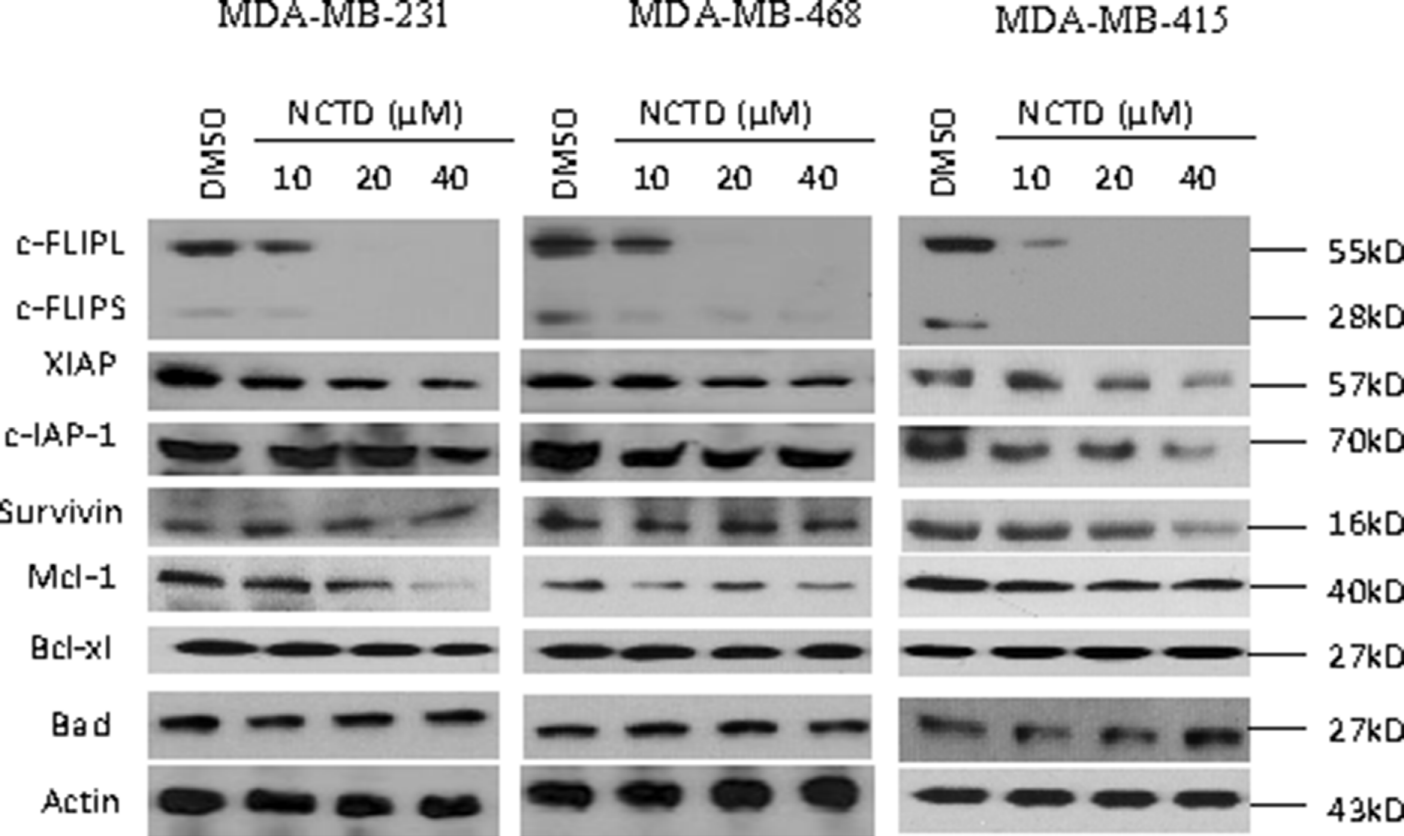
NCTD suppresses c-FLIP expression and Mcl-1 in breast cancer cells Breast cancer MDA-MB-231, MDA-MB-468, MDA-MB-415 cell lines were treated by SMAC mimetic Birinapant for 24 h. Cells were harvested and cell lysates were examined for the expression of c-FLIP, c-IAP-1, XIAP, Survivin, Mcl-1, Bcl-xl and Bad. Actin was used as a loading control.

We examined the effect of NCTD on the expression of a panel of critical apoptosis-related proteins, including c-FLIP, XIAP, c-IAP-1, Survivin, Mcl-1, Bcl-xl and Bad, in these breast cancer cell lines. We treated the cells with 10, 20 and 40 μM of NCTD for 48 h since CCK-8 assay showed treatment with this concentration-range of NCTD partially inhibited cell viability in these 4 cell lines (Supplementary Figure 1B). Western blotting analysis showed that NCTD effectively reduced the protein level of c-FLIPL and also the protein level of c-FLIPS in all four cell lines in a dose-dependent manner (Figure 2 and Supplementary Figure 1C). Specifically, NCTD at 10 μM partially inhibited the levels of c-FLIP proteins expression, while at 20 and 40 μM reduced c-FLIP proteins expression to negligible levels in these breast cancer cells. We next used qRT-PCR assay to investigate the effect of NCTD on the mRNA expression of c-FLIP in MDA-MB-231 and MDA-MB-468 cell lines treated by NCTD (Supplementary Figure 1D). We found that treatment with NCTD for 24 h, the mRNA level of c-FLIP was significantly inhibited in both cell lines, suggesting that the c-FLIP inhibition might be through a transcriptional mechanism. Interestingly, we noted that NCTD at 40 μM modestly decreased Mcl-1 level in MDA-MB-231 cell line, but not in other three cell lines. This Mcl-1 inhibition might be caused by the cytotoxic effect of NCTD at this high concentration in this cell line (Figure 2). In contrast, NCTD had no or little effect on the expression levels of other proteins in these breast cancer cell lines.

### NCTD sensitizes breast cancer cells to Birinapant-mediated cell viability assay

**Figure 3:**
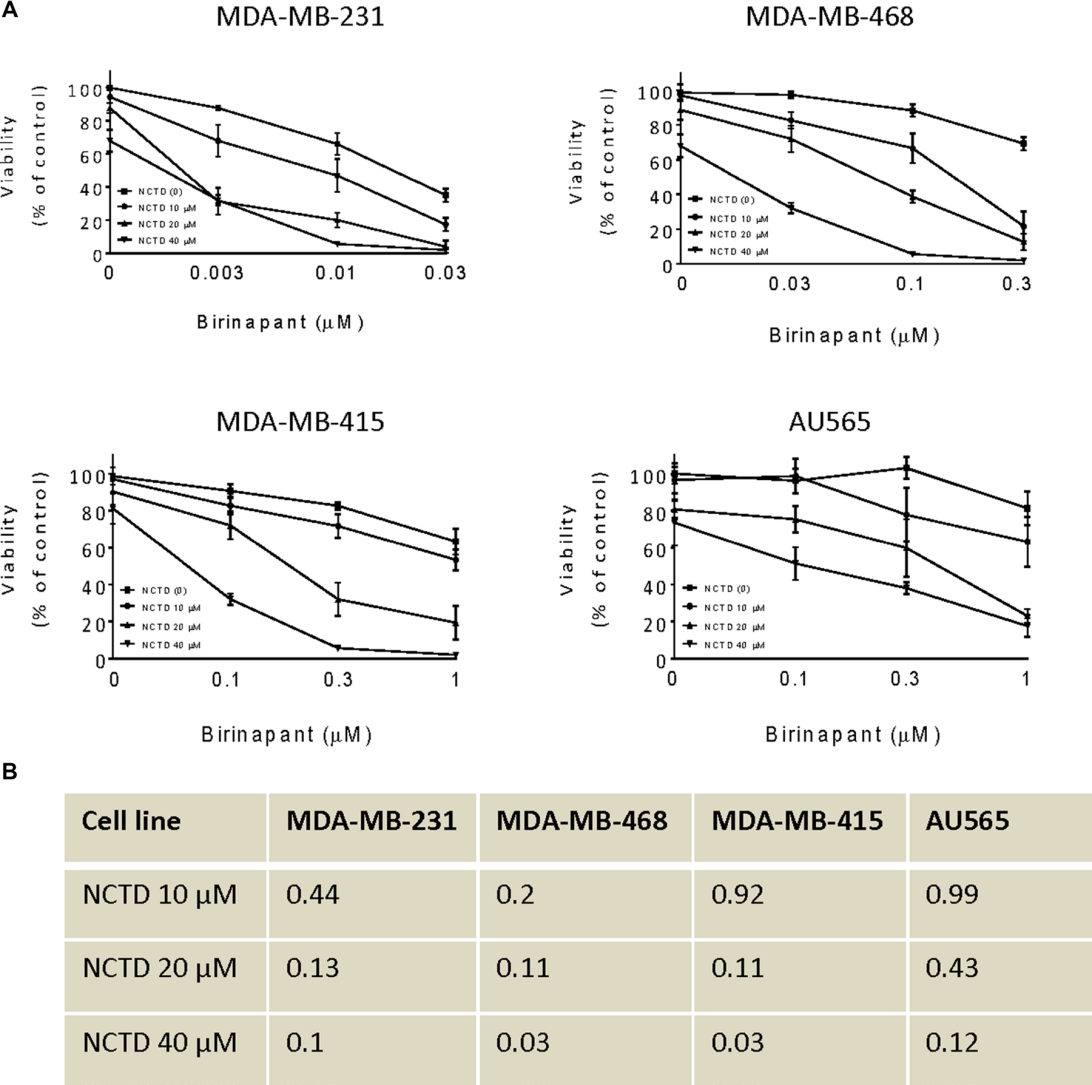
NCTD sensitizes breast cancer cells to Birinapant-mediated cell viability Human breast cancer MDA-MB-231, MDA-MB-468, MDA-MB-415 and AU565 cell lines were treated by Birinapant alone, NCTD alone, or both for 48 h. (**A**) cell viability was determined by CCK-8 assay. The data are representative results of three independent experiments. (**B**) The CI was calculated with an equation described in the Material and Method.

Since c-FLIP is a critical mediator of SMAC mimetic-resistance in cancer cells [15, 16], we hypothesized that suppression of c-FLIP by NCTD could sensitize breast cancer cells to Birinapant-mediated anticancer activity. We then examined the combinational effect of Birinapant with NCTD in these breast cancer cell lines by CCK8 assay. In MDA-MB-231 cell line, we found that Birinapantat at 10 nM inhibited cell viability by 34% in MDA-MB-231 cell line, whereas Birinapant in combination with NCTD at 10, 20 and 40 μM inhibited cell viability by 53, 80 and 97%, respectively. These results showed that NCTD further sensitized this SMAC mimetic-sensitive breast cancer cell line to Birinapant-mediated anticancer activity (Figure 3A). CCK8 assay further showed that NCTD also enhanced Birinapantat-activity in other 3 SMAC mimetic-resistant cell lines (Figure 3B). Combination index (CI) analysis revealed that the combination showed synergistic effect in all 4 breast cancer cell lines when Birinapantat was combined with NCTD at 20 and 40 μM, and showed synergistic or additive effect in all 4 cell lines when combined with NCTD at 10 μM. These results suggest that NCTD is able to convert SMAC mimetic-resistant cancer cell lines to sensitive ones (Figure 3B). Furthermore, it is noted that SMAC mimetic Birinapant as a signal-agent could not completely inhibit cell viability in these cancer cell lines (Figure 1, and solid squares in Figure 3A). Even in the most sensitive MDA-MB-231 cell line, approximately 10% of cells survived 48 h-treatment by 300 nM Birinapant (Figure 1A). However, in combination with NCTD, Birinapantat at much lower concentrations (10 nM for MDA-MB-231, 300 nM for MDA-MB-468 and 300 nM for MDA-MB-415) inhibited cell viability almost by 100% in the cells, indicating a strong cytotoxic effect by the combination.

### NCTD enhances Birinapant-mediated anticancer activity in breast cancer cells

**Figure 4:**
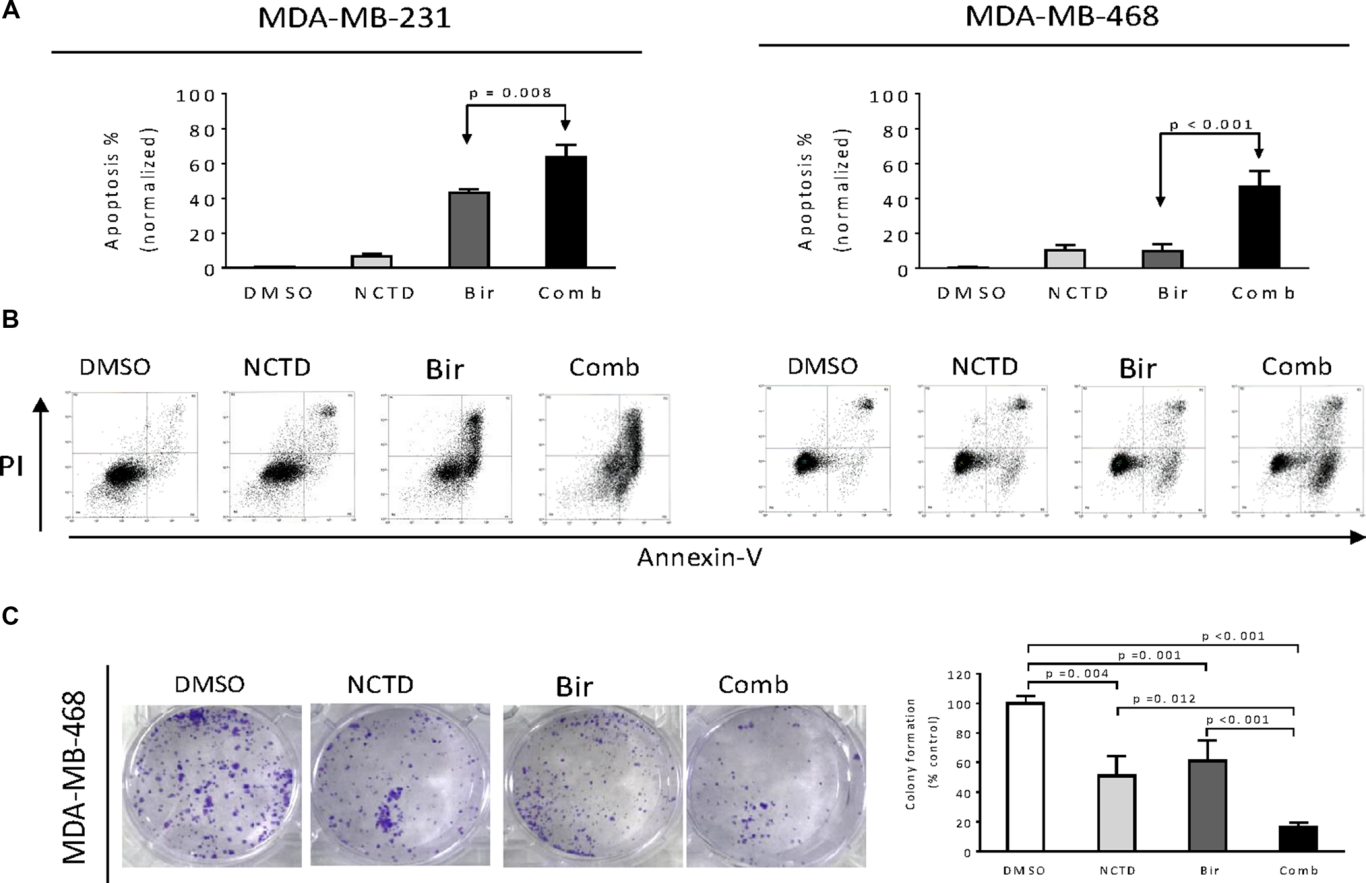
NCTD enhances Birinapant-mediated anticancer activity in breast cancer cells Breast cancer MDA-MB-231 and MDA-MB-468 cell lines were treated by Birinapant (Bir) alone, NCTD alone, or both (Comb) for 48 h, apoptosis was determined by Annxin-V-FITC/PI staining and flow cytometry assay. (**A**) Average results of three independent experiments were plotted. (**B**) One representative figure was shown for each treatment. (**C**) MDA-MB-468 cell line seeded in 6-well plates (1,000 cells per well) were treated by Birinapant (Bir) alone, NCTD alone, or both (Comb) for 12 days to allow colony formation. The colonies were stained with 0.05% crystal violet. (left panel) Representative figure was shown for each treatment, (right panel) average results of three independent experiments were plotted.

We used NCTD at 20 μM in this apoptosis assay because NCTD at this concentration markedly inhibited the protein levels of c-FLIPL and c-FLIPS expression but had minimal single-agent activity in these breast cancer cell lines. We treated MDA-MB-231 and MDA-MB-468 cell lines with Birinapant alone, NCTD alone or both for 48 h. We then examined apoptosis in the treated cells by Annexin-V/PI staining and flow cytometery assay. In MDA-MB-231 cell line, we found that after normalized to DMSO control, Birinapantat at 20 nM alone induced apoptosis in 40% cells, and NCTD had weak apoptotic effect. In contrast, the combination induced apoptosis in about 67% cells, showing the strong sensitization of Birinapant-mediated apoptosis by NCTD (left panel, Figure 4A, 4B). In MDA-MB-468 cell line, we found that Birinapant at 100 nM or NCTD at 20 μM alone induced apoptosis only in approximately 10% cells. In striking contrast, their combination induced apoptosis in approximately 50% cells (right panel, Figure 4A, 4B), consistent with strong inhibitory effect on cell viability by the combination in this cell line.

We next performed colony formation assay in MDA-MB-468 cell line and found that either single agent could reduce the colony formation as compared to DMSO treatment. In contrast, their combination had much stronger activity in reducing colony formation than either single agent. These results suggest that the combination have greater inhibitory effect on long-term growth of breast cancer cells (Figure 4C).

### Caspase activity plays an essential role in apoptosis mediated by the combination in breast cancer cells

**Figure 5:**
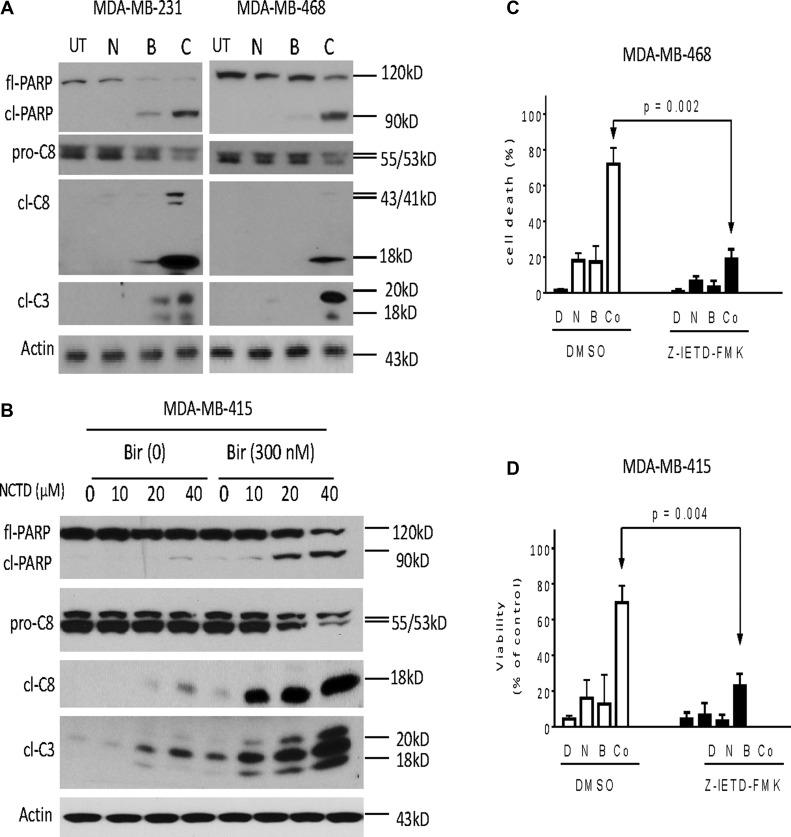
Caspase activity plays a critical role in apoptosis induced by Birinapant in combination in breast cancer cells Breast cancer (**A**) MDA-MB-231, MDA-MB-468 and (**B**) MDA-MB-415 cell lines were treated by Birinapant (Bir) alone, NCTD alone, or both (Comb) for 24 h. Treated cells were harvested and cell lysates were examined for the expression of full-length PARP (fl-PARP), cleaved PARP (cl-PARP), pro-Caspase-8 (pro-C8), cleaved Caspase-8 (cl-C8) and cleaved Caspase-3 (cl-C3). Actin was used as a loading control. The data are representative results of three independent experiments. (**C**) MDA-MB-468 and (**D**) MDA-MB-415 cell lines pretreated with 50 μM caspase-8 inhibitor Z-IETD-FMK for 1 h were treated by 0.3 μM Birinapant alone, 20 μM NCTD alone or both for 48 h, cell death induction was determined with trypan blue exclusion assays. *p < 0.05, **p < 0.01.

We next performed western blotting analysis to dissect major apoptosis signaling pathways triggered by Birinapant alone, NCTD alone or both in three cell lines. Because the apoptosis signaling activation is an early event, we treated the breast cancer cells for 24 h. In MDA-MB-231 cell line, treatment with Birinapant at 20 nM alone induced modest accumulation of proteolytic activated bands of initiator caspase-8 and executioner caspase-3, as well as accumulation of cleaved poly (ADP-ribose) polymerase (PARP). Addition of NCTD noticeably increased these actions, consistent with the aforementioned results that NCTD enhanced Birinapant-mediated apoptosis in this SMAC mimetic-sensitive cell line (Figure 5A). In MDA-MB-468 cell line, treatment with signal agents had no or minimal effect on activation of caspase-8, -3, and PARP cleavage. In striking contrast, their combination induced robust accumulation of cleaved PARP and activated caspase-8, -3, accompanied by marked decreases of pro-caspase-8, pro-caspase-3, as well as decreases of full-length PARP (fl-PARP) (Figure 5A). Dose-dependently enhanced activation of caspase-8, caspase-3 and PARP cleavage were noted in MDA-MB-415 cell line (Figure 5B). Additionally, the combination had no effect on the activation of caspase-9, an initiator caspase for intrinsic apoptosis (data not shown).

Moreover, SMAC mimetics-mediated anticancer activity primarily depends on TNFα-triggered caspase-8-dependent extrinsic apoptosis pathway [6, 7]. We thus investigated whether the enhancement of Birinapant-mediated anticancer activity by NCTD in breast cancer cells was through a similar mechanism. In this regard, we first pretreated MDA-MB-231 and MDA-MB-468 cells with Z-IETD-FMK, a specific caspase-8 inhibitor for 1 h, and then treated the cells by combination for another 48 h. We found that cell death induction by the combination was significantly attenuated by the caspase-8 inhibitor (Figure 5C, 5D). We next pretreated with neutralizing antibodies (2 μg/ml) against TNFα and TNF-related apoptosis-inducing ligand (TRAIL) for 2 h, and then treated with the combination for another 48 h. We found that cell death induction by the combination was effectively blocked by the TNFα, but not by the TRAIL antibodies in both cell lines (Supplementary Figure 2), suggesting that apoptosis induction by the combination is triggered by TNFα.

### NCTD enhances Birinapant-mediated cell death induction in primary breast cancer cells

**Figure 6:**
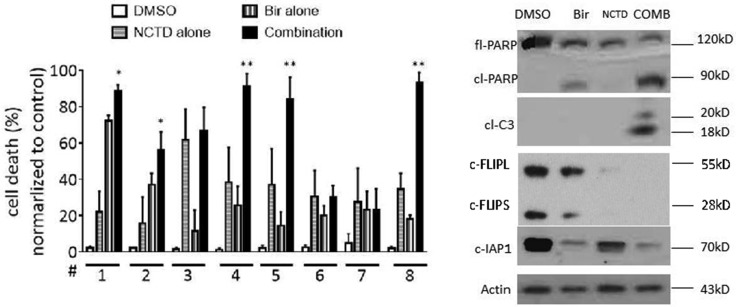
NCTD enhances Birinapant-mediated cell death induction in primary breast cancer cells (**A**) Primary breast cancer cells isolated from 8 freshly surgically resected breast tumors were treated with Birinapant at 0.1 μM alone, NCTD at 20 μM alone or both for 48 h, cell death induction was determined with trypan blue exclusion assays. *p < 0.05, **p < 0.01. (**B**) Primary breast cancer cells from No.5 case were treated with Birinapant at 0.1 μM alone, NCTD at 20 μM alone or both for 48 h. The expression levels of cIAP-1, PARP, Caspase-3 and c-Flip were examined by western blotting analysis. Actin was used as a loading control.

We further evaluated the response of primary breast cancer cells to NCTD alone, Birinapant or the combination to explore the clinical relevance of this combination strategy. Primary breast cancer cells freshly isolated from surgically resected tumor tissues of 8 female patients were tested. The mean tumor size was 35 ±14 mm (ranging 15 mm to 58 mm) (Supplementary Figure 3). No patients received chemotherapy before operation. After single cell suspension isolation, breast cancer cells were treated by 20 μM NCTD alone, 0.1 μM Birinapant alone, or their combination for 48 h, and analyzed for cell death by trypan blue assay. We found that Birinapant induced obvious cell death only in 1 primary breast cancer cells, while had no or modest effect in other 7 primary breast cancer cells. Moreover, NCTD alone had little or no effect in these primary cells. In contrast, the combination effectively triggered massive cell death in the primary breast cancer cells from No.5 case. Of note, as compared to either single-agent treatment, the combination effect in primary cancer cells from this case was significantly improved (*p <* 0.05) (Figure 6B). Western blotting showed that NCTD markedly reduced the level of c-FLIP and enhanced Birinapant-triggered caspase-3 activation and PARP cleavage in primary breast cancer cells of case 5, suggesting a similar mechanism as in established cancer cell lines (Figure 6B).

## DISCUSSION

Small molecule SMAC mimetics are newly developed anticancer agents. Preclinical studies demonstrated that SMAC mimetics potently induced apoptosis in certain types of cancers and effectively inhibited tumor growth in xenograft models, suggesting that SMAC mimetics hold promise for human cancer patients. Nevertheless, clinical trials showed that resistance to the single-agent treatment of SMAC mimetics was very common among cancer patients, posting a serious problem for the potential clinical application, and calling for novel strategies to improve SMAC mimeitc-efficacy [10, 18–20]. In the present study, we discover a combination approach that can be useful in promoting the efficacy of SMAC mimetic-based breast cancer treatment. This combination approach is composed of SMAC mimetic Birinapant and NCTD, a major bioactive constituent of Traditional Chinese Medicine blister beetle Mylabris. Since Birinapant is a drug candidate that currently is under clinical evaluation for human cancer treatment, and NCTD is used clinically as a conventional anticancer drug, our discovery has important clinical relevance and warrants further preclinical and clinical investigations. Moreover, the importance of our discovery is further underscored by our findings that the potent anticancer activity was observed in 3 triple-negative MDA-MB-231, MDA-MB-468 and AU565 cell lines, suggesting that this combination strategy might offer a treatment option for this lethal form of breast cancer.

Mechanistic studies revealed that extrinsic apoptosis is responsible for the enhancement of Birinapant-mediated anticancer activity by NCTD in breast cancer cells. We reached this conclusion by several pieces of evidences. Firstly, western blotting analysis of apoptosis signaling showed that as compared to single-agent treatments, the combination distinctly amplified caspase-8-caspase-3 cascade in cancer cells in both cell lines, which led to more cleavage of DNA repair enzymes PARP. Secondly, inhibition of initiator caspase-8 by Z-IETD-FMK significantly attenuated the cell death induction by the combination, suggesting an essential role of extrinsic pathway in the combination-mediated apoptosis. Thirdly, the strong anticancer activity by combination could be significantly inhibited by a TNFα-, but not by a TRAIL- neutralizing antibody, indicating that activation of apoptosis signaling was triggered by TNFα secreted by the cancer cells in an autocrine fashion.

C-FLIPL and c-FLIPS are oncoproteins that have drawn attention in the field of cancer targeted therapy recently. Increased expression of c-FLIPL and c-FLIPS was recorded in cell lines from breast cancer and many other cancer cells [15, 21–23]. Another key finding in this study is that NCTD potently inhibits the expression of two isoforms of c-FLIPL and c-FLIPS in breast cancer cells. This finding is in agreement with previous observation in esophageal cancer cells, suggesting that this inhibitory effect by NCTD is not limited to single cell type. RT-PCR analysis indicates that the inhibitory effect on c-FLIPL and c-FLIPS is mediated by a transcriptional mechanism. Because both c-FLIPL and c-FLIPS exhibit the oncogenic function primarily by inhibiting caspase-8 activation, our data thus indicate that the inhibition of c-FLIPL and c-FLIPS may be responsible for enhancement of caspase-8 activation by the combination.

## MATERIALS AND METHODS

### Cell lines and compounds

Human breast cancer cell lines MDA-MB-231, MDA-436, MDA-415, AU565 were purchased from Cell Bank of the Chinese Academy of Sciences Shanghai Institute of Cell Biology (Shanghai, China) and maintained in RPMI 1640 (HyClone/Thermo Fisher Scientific, Beijing, China) supplemented with 10% heat-inactivated fetal bovine serum (Hangzhou Sijiqing Biological Engineering Materials Co., Ltd, Hangzhou, China). Cells were incubated in a humidified 5% CO2 atmosphere at 37°C. Birinapant and Z-IETD-FMK were purchased from Selleck (Shanghai, China) and was dissolved in DMSO with a stock concentration of 1 and 5 mmol/L and stored at −20°C. NCTD was purchased from Nanjing Zelang Medical Technology Co., Ltd (Nanjing, China), and was dissolved in distill water with a stock concentration of 2 mM.

### Primary cells from breast cancer tissues

Tissue pieces from breast cancer patients were collected during surgery. Informed written consent was obtained from each patient for the use of individual patient, and the study has been approved by the Hospital Institutional Review Board of the First Hospital of TsingHua University. The tissue samples were cut into small blocks of approximately 1 mm^3^ and washed extensively in PBS. Then tissue pieces were dissociated with trypan and collagenase type IV into signal cells for further experiments.

### Cell counting kit-8 (CCK-8) cell proliferation cytotoxicity assay

Breast cancer cells seeded in triplicate in microtiter plates (96 wells) with a density of 3 × 10^3^ cells per well in 100-μl medium were treated as indicated in the text and figure legends for 72 h. The CCK-8 reagent (Roche, Shanghai, China) was added to cells at a concentration of 10 μL/well in 100 μL culture medium and the samples were incubated at 37°C for 2–4 hours. The optical density (OD) of each sample was measured using a microplate reader at 450 nM. The percentages of absorbance relative to those of untreated control samples were plotted as a linear function of drug concentration. Inhibition of cell viability was measured by percentage of viable cells relative to the control: % inhibition = 100% × ODT/ODC, where ODT is the average OD value of the treated samples and ODC is the average OD value of the control samples. Combination index (CI) was calculated by an equation reported in a previous report [24]. CI less than 0.9 indicates synergism; 0.9 to 1.1, additivity; and greater than 1.1, antagonism.

### Analysis of apoptosis

Apoptosis was examined using Roche Annexin-V-FLUOS Staining Kit (Haoran, Shanghai, China) according to the manufacturer's instructions. Before flow cytometric analysis, cells were harvested and resuspended in 500 μl binding buffer, the cell suspension was added to Annexin V/FITC (5 μl) and homogeneous mixing, 5 μl of Propidium Iodide (PI) was added to the solution and the samples were incubated at room temperature in the dark for 15 min. Each sample containing 1–3 × 10^5^ cells were measured using a BD LSR II system (BD Biosciences) analyzed by the DiVA software (version 4.1.2; BD Biosciences).

### Colony formation assay

For the colony formation assay, 1,000 MDA-MB-468 cells were seeded in a 6-well plate and incubated with 20 μM NCTD, 5 μM Birinapant or a combination of the two for 12 days. The cell colonies were fixed with methanol for 5 min at room temperature and then stained with 0.05% crystal violet in 50% methanol and 10% glacial acetic acid for counting. Colonies with a diameter > 0.2 mm were counted and the results were presented as a histogram. Colony formation experiments were performed in triplicate and repeated in three independent experiments.

### Western blotting

Western blotting analyses were performed as described previously [12]. Antibodies used were as follows: anti-cFLIP (G-11) mouse monoclonal antibody (sc-5276); anti-Actin goat polyclonal antibody, HRP-conjugated secondary anti-mouse, anti-goat and anti-rabbit antibodies from Santa from Santa Cruz Biotechnology (Shanghai, China); anti-cIAP-1 (AF8181) from R&D Systems (Shanghai, China); anti-caspase-8 mouse monoclonal antibody (9746), anti-caspase-3 rabbit polyclonal antibody (9665) and anti-PARP (9542) from Cell Signaling Technology.

### Quantitative real-time PCR

Total cellular RNA was extracted from cancer cells using TRIzol reagent (Invitrogen, Shanghai, China). For first-strand complementary DNA synthesis, 1 μg total RNA was reverse transcribed using the High Capacity cDNA kit (Applied Biosystems Trading Shanghai Co Ltd, Shanghai, China) according to the manufacturer's instructions. For PCR, the primer sequences and expected product sizes were as follows: c-FLIP (512 bp), forward: 5′-ATGTCTGCTGAAGTCAT CC-3′, reverse: 5′-ATCCTC ACCAATCTCCTGCC-3′; β-actin (475 bp), forward: 5′-TG ACGGGGTCACCCACACTGTGCC-3′, reverse: 5′-CTG CATCCTGTCGGCAATGCCAG-3. Real-time PCR was performed as described previously [12]. Data are presented as relative quantity normalized to the housekeeping gene β-actin.

### Statistical analysis

At least three separate experiments were performed. One-way ANOVA and Student *t* tests were used to determine significance between experimental groups. A *p* value < 0.05 was considered significant.

## SUPPLEMENTARY MATERIALS FIGURES AND TABLES


