# Supplementary material for: NCTD promotes Birinapant-mediated anticancer activity in breast cancer cells by downregulation of c-FLIP
Source: Oncotarget. 2017 Mar 2;8(16):26886–95. doi: 10.18632/oncotarget.15848 (PMC5432304; doi:10.18632/oncotarget.15848)
Supplement: Supplementary file 1 [file oncotarget-08-26886-s001.pdf]

NCTD promotes Birinapant-mediated anticancer activity in breast cancer cells by downregulation of c-FLIP

Supplementary Materials

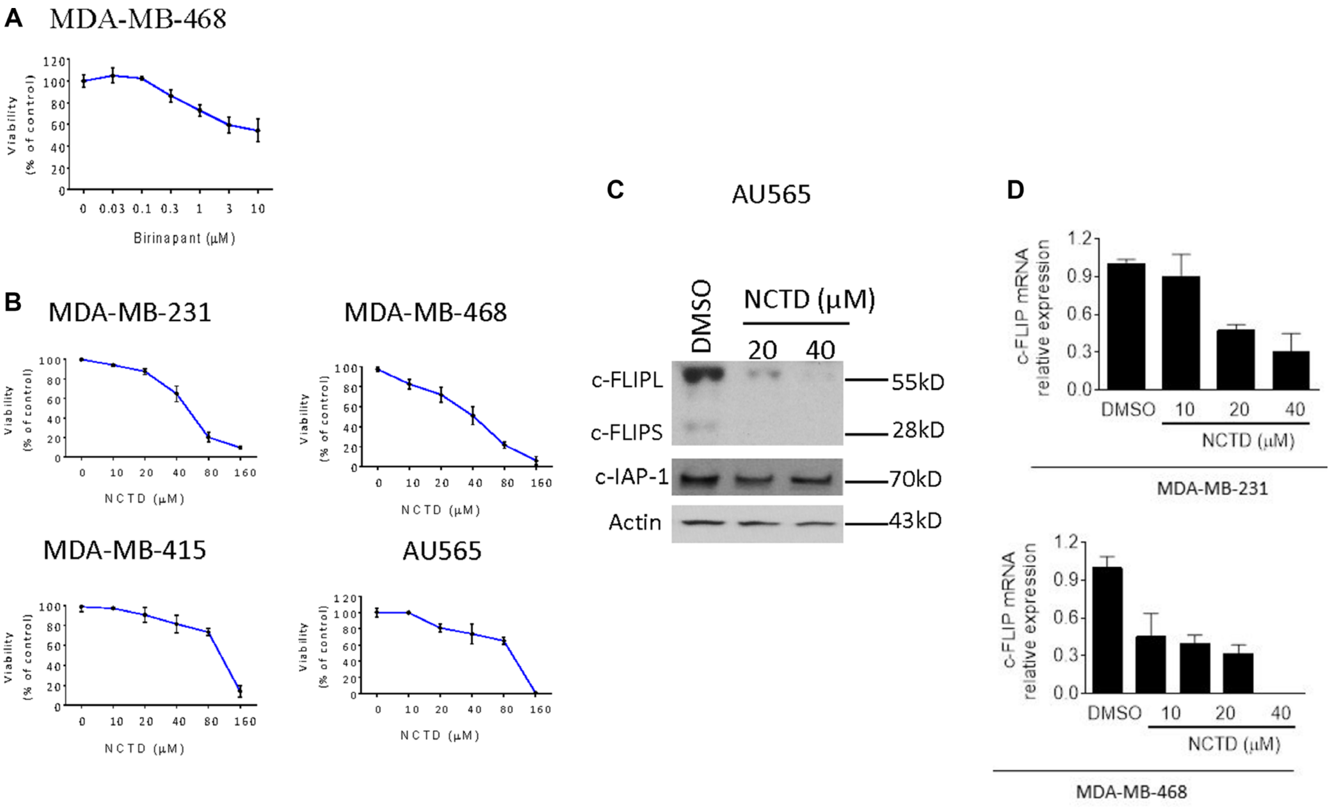

Supplementary Figure 1: Single agent activity of NCTD in breast cancer cells. (**A**) MDA-MB-468 cell line were treated with Birinapant for 4 days, cell viability was determined by CCK-8 assay. (**B**) Breast cancer cell lines MDA-MB-231, MDA-MB-468, MDA-MB-415 and AU565 were treated by NCTD for 48 h, cell viability was determined by CCK-8 assay. (**C**) AU565 cells were treated by NCTD at 20 and 40 µM for 48 h and the expression of c-FLIPL, c-FLIPS and c-IAP-1 was examined by western blotting analysis. Actin was used as a loading control. (**D**) MDA-MB-231 and MDA-MB-468 cell lines were treated by NCTD for 24 h. Total RNA was isolated and the mRNA level of c-FLIP was were examined by quantitative Real-Time PCR assay. The results are the averages of three independent experiments.

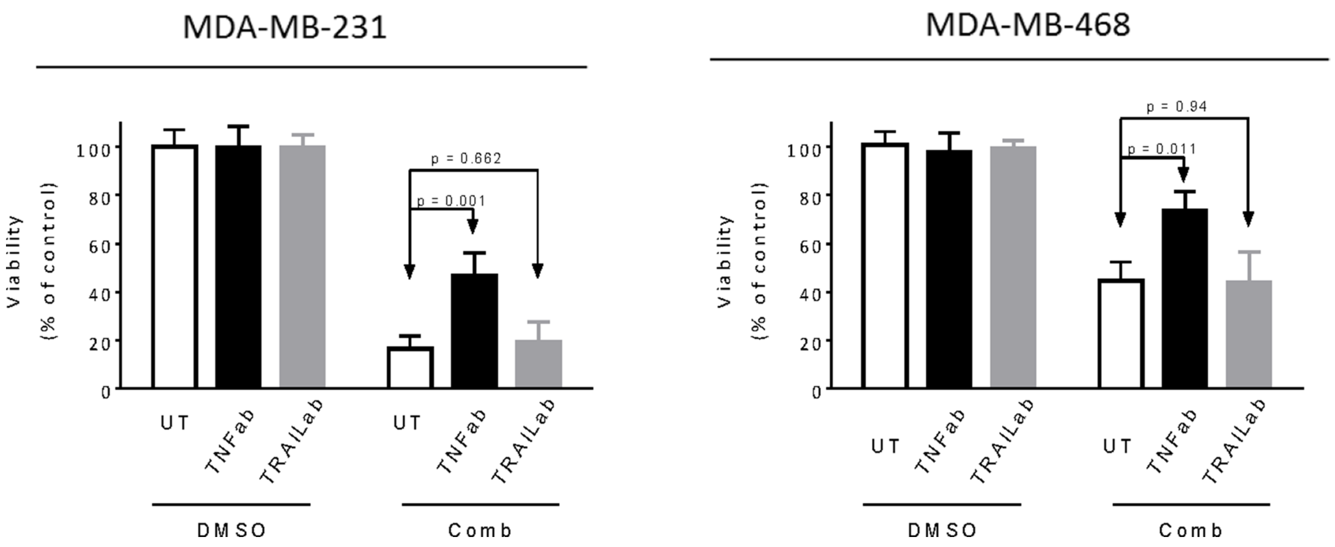

Supplementary Figure 2: TNFα plays an important role in the combination. Human breast cancer MDA-MB-231 and MDA-MB-468 cell lines pretreated with TNF neutralizing antibody or TRAIL neutralizing antibody for 1 h, were treated by Birinapant alone, NCTD alone, or both for 48 h, cell viability was determined by CCK-8 assay.

Clinical pathological characteristics of breast cancer patients

Case No.	Age (years)	Tumor volume (cm X cm)	local metastasis	Distance metastasis	ER status	PR status	Her2
1	35	4.5X1.7	(-)	no	(+)	(+)	(+)
2	46	3.3X1.8	(+)	no	(+)	(+)	(-)
3	38	4.4X2.1	(+)	no	(-)	(-)	(-)
4	42	2.9X2.4	(-)	no	(+)	(+)	(-)
5	28	3.7X2.0	(-)	liver	(-)	(-)	(-)
6	33	1.5X1.3	(-)	no	(+)	(+)	(-)
7	47	5.8X2.5	(+)	no	(+)	(-)	(+)
8	39	1.8X1.6	(+)	lung	(-)	(-)	(-)

Supplementary Figure 3: Clinical pathological characteristics of breast cancer patients. No other Figure legend for Supplementary Figure 3
